# Reliable Alignment in Total Knee Arthroplasty by the Use of an iPod-Based Navigation System

**DOI:** 10.1155/2016/2606453

**Published:** 2016-05-30

**Authors:** Paola Koenen, Marco M. Schneider, Matthias Fröhlich, Arne Driessen, Bertil Bouillon, Holger Bäthis

**Affiliations:** ^1^Department of Orthopaedics, Trauma Surgery and Sports Medicine, Cologne Merheim Medical Center, Witten/Herdecke University, 51109 Cologne, Germany; ^2^Department of Orthopaedic Surgery, Schulthess Clinic, 8008 Zurich, Switzerland

## Abstract

Axial alignment is one of the main objectives in total knee arthroplasty (TKA). Computer-assisted surgery (CAS) is more accurate regarding limb alignment reconstruction compared to the conventional technique. The aim of this study was to analyse the precision of the innovative navigation system DASH® by Brainlab and to evaluate the reliability of intraoperatively acquired data. A retrospective analysis of 40 patients was performed, who underwent CAS TKA using the iPod-based navigation system DASH. Pre- and postoperative axial alignment were measured on standardized radiographs by two independent observers. These data were compared with the navigation data. Furthermore, interobserver reliability was measured. The duration of surgery was monitored. The mean difference between the preoperative mechanical axis by X-ray and the first intraoperatively measured limb axis by the navigation system was 2.4°. The postoperative X-rays showed a mean difference of 1.3° compared to the final navigation measurement. According to radiographic measurements, 88% of arthroplasties had a postoperative limb axis within ±3°. The mean additional time needed for navigation was 5 minutes. We could prove very good precision for the DASH system, which is comparable to established navigation devices with only negligible expenditure of time compared to conventional TKA.

## 1. Introduction

Joint replacement surgery has been established as a standard therapy for severe osteoarthritis of the knee. However, there are still a reasonable number of revision surgeries. One of the most important reasons for arthroplasty failure is aseptic loosening [1, 2]. Despite a current debate on natural alignment, malalignment must still be considered as one of the main reasons for aseptic loosening [3, 4]. Earlier studies have shown that alignment in the coronal plane within the range of 3° varus/valgus is associated with better survival of the prosthesis [5–9], which cannot be achieved by the conventional technique in up to 30% [3, 5]. In this context, computer-assisted surgery (CAS) has found its way into joint replacement surgery. Since the first report about CAS in total knee arthroplasty (TKA) in 1999 [10], multiple navigation systems have been developed. Considering various meta-analysis from different groups, CAS in total knee replacement has proven to be more accurate regarding limb alignment reconstruction as well as component placement compared to the conventional technique without doubt [5, 9, 11]. However, several reasons have to be considered as to why the navigation technique is still not regularly used besides its valuable advantages. One of the main obstructions for the technique is additional costs resulting from direct costs for investment and indirect costs such as additional time for the surgical procedure. Furthermore, the computer-assisted technique is still perceived as a difficult procedure with a prolonged training curve for new users [7, 11]. Based on these conditions, we were involved in development cooperation of a new image-free navigation system in order to increase the acceptance for the navigation technique. The main intention was to develop a navigation system with simplified user-friendly workflow, intuitive handling, and optimized time frame for the setup of the system as well as for the surgery itself.

The aim of this study was to analyse the precision of this innovative navigation system called DASH, which has previously been described as a “smart mobile solution” [12].

## 2. Materials and Methods

### 2.1. Navigation System

**Figure 1:**
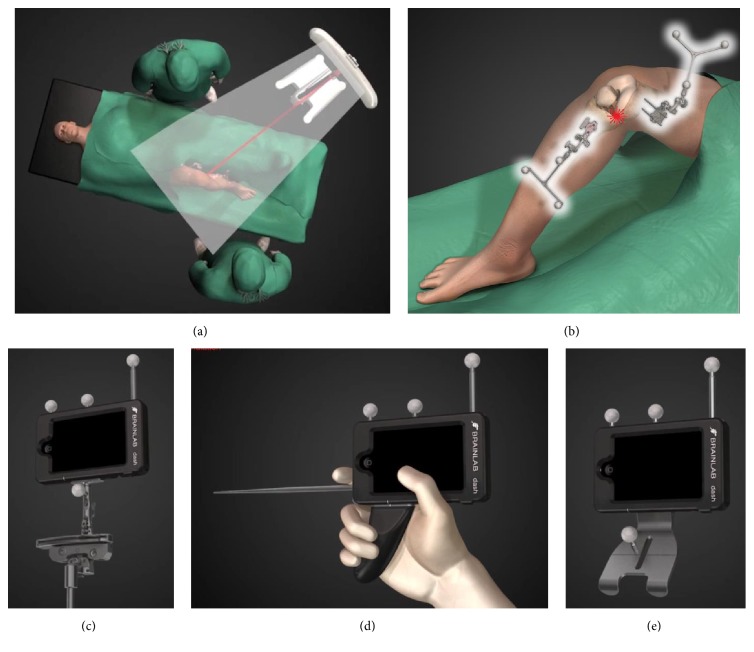
(a) Intraoperative setting. (b) Reference arrays attached to femur and tibia. (c) The cradle is attached to the cutting block for proximal tibial resection. (d) Digitalisation of anatomical landmarks performed using the pointer attached to the handheld cradle. (e) Digitalisation of distal femur anatomy is performed using a special digitalisation tool attached to the cradle.

**Figure 2:**
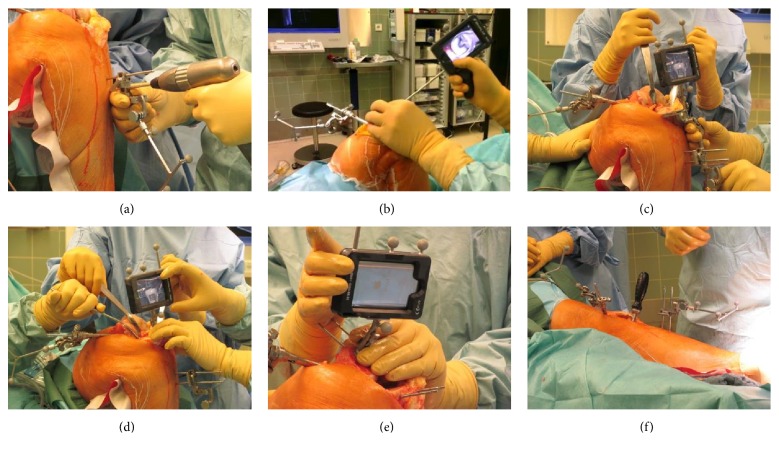
(a) Attachment of reference arrays. (b) Digitalisation of anatomical landmarks using the pointer, attached to the handheld cradle. (c) The cradle is attached to the cutting block for proximal tibial resection. Data is shown on the iPod screen (varus/valgus, resection level, and slope). (d) Verification of tibial resection. (e) Verification of distal femoral resection. (f) Control of leg alignment and ligament situation.

The DASH Navigation System by Brainlab (Munich, Germany) is designed as an image-free navigation system. All joint information is digitised during surgery without the need for additional preoperative diagnostics. Central to the hardware concept is a sterile draped Apple iPod Touch® that is included in a handheld cradle and serves as the operating unit. The iPod works remotely with the separated computer platform that is included in the infrared-camera stand using secured wireless LAN connection. Additional instruments are attached to the handheld cradle that is equipped with three reflecting marker spheres, which are tracked by the infrared camera. During navigation assisted bone resection and cut verification, the iPod cradle is attached to the universal resection guides of the knee system or on top of the final bone resection plane. The principles of the workflow are comparable to the established navigation technology in TKA. After skin and capsular incision, reference arrays are attached to the distal femur. Afterwards, digitalisation of a number of anatomic landmarks is necessary, which is performed using a pointer, attached to the handheld cradle. The following landmarks have to be digitised: distal endpoint of the femoral mechanical axis, Whiteside line, surface of the medial and lateral condyle, insertion of the anterior cruciate ligament, medial and lateral tibial plateau surface, and AP direction of the tibia as well as the medial and lateral malleolus. The centre of the femoral head is digitised by hip pivoting. Afterwards, the software can be used, as preferred by the surgeon, on the femur or tibia first and the surgeon is able to go back to the other resection at any time. For resection, the iPod cradle is attached to the cutting block and positioned using the iPod screen. The surgeon gets instant and comprehensive information on the resection level, femoral coronal and flexion alignment, and tibial coronal and slope alignment, which is visible directly in the surgical field. Verification of rotational orientation of the femoral component is not supported by the system and was determined ligament-balanced using conventional spacer blocks. After the bone cut, the resection plane can be verified with the same technique and the results are saved for the final patient report. For details of the surgical procedure, see Figures 1 and 2.

### 2.2. Study Design

A retrospective analysis of 40 consecutive computer-assisted total knee arthroplasties (PFC Sigma, DePuy) was performed. TKA was performed by a surgeon with extensive experience in CAS TKA. Intraoperatively acquired navigation data were documented.

Axial limb alignment was evaluated on pre- and postoperative full-length weight-bearing radiographs. Radiographs were performed using an internal standardized protocol according to the recommendations described by Cooke et al. [13]. To avoid errors due to limb rotation or knee flexion, postoperative radiographs were delayed until full knee extension was achieved.

**Figure 3:**
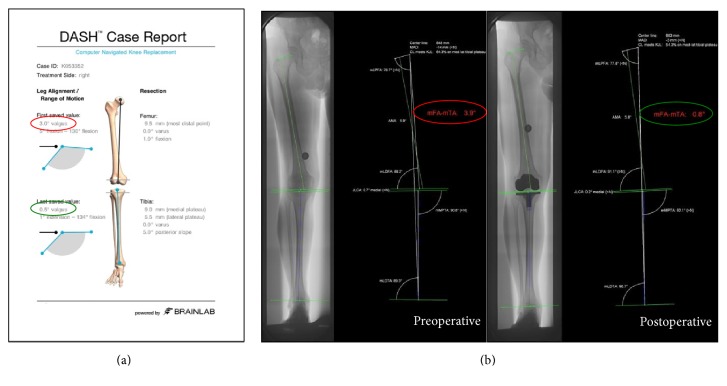
(a) Patient report generated by the navigation system after surgery. (b) Pre- and postoperative full-length weight-bearing radiographs. Axial limb alignment was measured using the planning tool mediCAD.

Measurements of mFA-mTA (mTFA, mechanical tibiofemoral angle), mLDFA (mechanical lateral distal femoral angle), and mMPTA (mechanical medial proximal tibial angle) were performed by two independent observers using the digital planning software mediCAD version 2.20 (Hectec, Niederviehbach, Germany). These data were compared with the intraoperatively acquired data of the navigation system (see Figure 3).

Interobserver reliability for measuring pre- and postoperative mFA-mTA, mLDFA, and mMPTA on radiographs was analysed using Pearson's correlation coefficient and evaluated according to Landis and Koch [14].

Furthermore, we used Pearson's correlation to evaluate the association between radiographic and navigation alignment measurements.

The length of the surgical procedure was documented for each patient and OR (operating room) time was compared to that of a control group including 125 conventional primary TKA performed in 2012 by the same surgical team. OR times of both groups were compared using unpaired Student's *t*-test; a *p* value < 0.05 was considered significant.

## 3. Results

40 computer-assisted primary total knee replacements were included. 13 patients were male and 27 female. Their mean age was 69 ± 7 years ranging from 53 to 84 years. 24 patients had surgery on the right knee and 16 on the left knee. The preoperative mechanical axis measured by X-ray varied between 20.3° varus and 18.4° valgus and the postoperative mechanical axis varied between 3.7° varus and 4° valgus, respectively.

**Figure 4:**
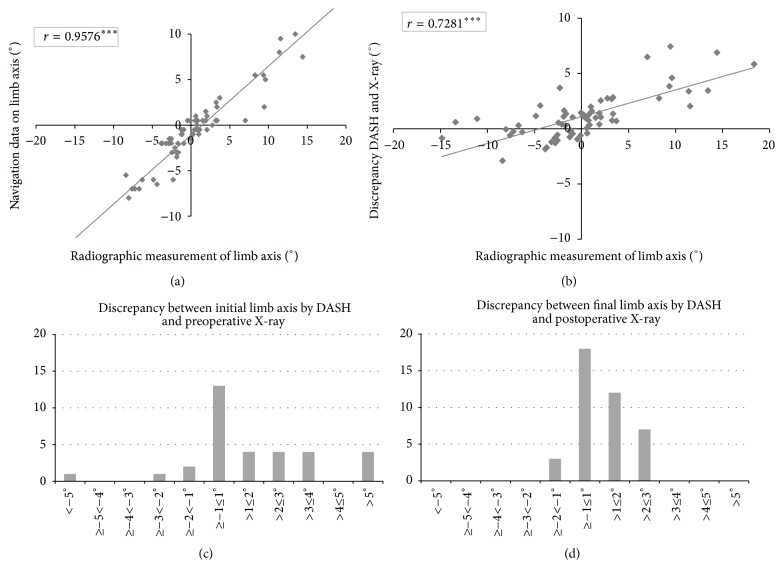
(a) Correlation between radiographic and navigation alignment measurements, shown as Pearson's correlation. ^*∗∗∗*^ *p* < 0.0001. (b) Correlation between limb axis by X-ray and discrepancy between radiographic and navigation measurements, shown as Pearson's correlation. ^*∗∗∗*^ *p* < 0.0001. (c) Distribution of the discrepancy between the preoperative mechanical axis by X-ray and the first intraoperatively measured limb axis by the navigation system. *n* = 40. (d) Distribution of the discrepancy between the postoperative mechanical axis by X-ray and the last intraoperatively measured limb axis by the navigation system. *n* = 40.

Our results show a strong correlation between radiographic and navigation alignment measurements (see Figure 4(a)) with Pearson's correlation coefficient of *r* = 0.96, which is rated “almost perfect” according to Landis and Koch [14]. However, the mean difference between the preoperative mechanical axis by X-ray and the first intraoperatively measured limb axis by the navigation system was 2.4°  ± 2.4°, whereas the postoperative X-rays showed only a mean difference of 1.3°  ± 0.9° compared to the final measurement of the navigation system, ranging from 0.1° to 9° and 0.1° to 3°, respectively. Distribution of the discrepancy between the mechanical axis by X-ray and the intraoperatively measured limb axis by the navigation system is shown in Figures 4(c) and 4(d) for the preoperative and postoperative setting. The discrepancy between radiographic and navigation measurements for limb axis increased with leg deformity (see Figure 4(b)).

According to our radiological measurements, 88% of arthroplasties had a postoperative limb axis within the range 3° varus/valgus; according to the navigation system, 100% were in this range. The mean postoperative mLDFA and mMPTA were 89.9°  ± 1.7° and 89.9°  ± 2.0°, respectively.

**Figure 5:**
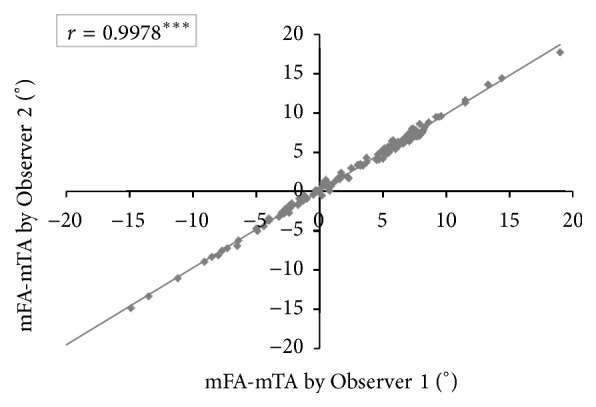
Interobserver reliability. mFA-mTA was measured on standardized full-length weight-bearing radiographs by two independent observers (Observer 1 and Observer 2) and Pearson's correlation coefficient (*r*) was calculated (^*∗∗∗*^ *p* < 0.0001).

Interobserver reliability was almost perfect with Pearson's correlation coefficients from 0.9356 to 0.9991 for calculating mFA-mTA, mLDFA, and mMPTA pre- and postoperatively between the two observers.  Figure 5 shows interobserver reliability for preoperative and postoperative measurements of mFA-mTA.

**Figure 6:**
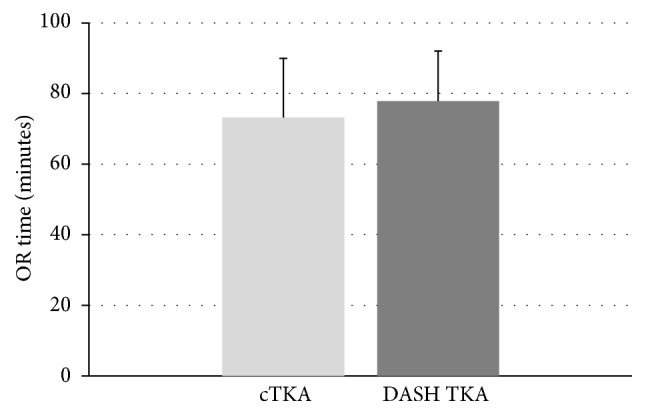
Duration of surgery for conventional TKA (cTKA, *n* = 125) and TKA using the navigation system DASH (DASH TKA, *n* = 40). Means + SD are shown (*p* = 0.12 by Student's *t*-test).

The mean duration of surgery was 78 ± 14 minutes in the navigation group compared to 73 ± 17 minutes in the conventional group, which was statistically not significant (*p* = 0.12 by Student's *t*-test). Results are shown in Figure 6.

## 4. Discussion

A main goal of this study was to compare the data of the new navigation system to our routine radiological long-leg X-ray technique which is still defined as the gold standard. The postoperative discrepancy between X-ray and navigation data was lower than between navigation and preoperative X-ray data. According to our radiological measurements, 88% of arthroplasties had a postoperative limb axis within the range 3° varus/valgus, whereas, according to the navigations systems, 100% were in this range. A meta-analysis by Cheng et al. showed that performing CAS TKA a limb axis within a range of 3° varus/valgus could be achieved in 87.8% [5]. Therefore, we could demonstrate that the precision of the DASH system is similar to established navigation systems.

12% of arthroplasties show a limb axis within ±3° measured by the DASH system but were out of this safe zone by radiographic measurement. This might be due to measuring differences between the two techniques. Several factors might limit the correlation between the two measurements: differences between both techniques on the one hand and sources of error of the single techniques on the other hand. Intraoperative measurements by navigation systems are made without weight-bearing and after skin and capsular incision while radiographic measurements are performed on weight-bearing X-rays without the surrounding tissue opened [15].

Different studies have further pointed out measuring inaccuracies on long-leg X-ray due to limb deformity [15], rotation of the leg, or flexion contracture [16]. Therefore, right patient positioning during X-ray is pivotal. In our study, radiographs were performed using an internal standardized protocol according to the recommendations described by Cooke et al. [13]. To avoid errors due to limb rotation or knee flexion, postoperative radiographs were delayed until full knee extension was achieved [17]. Nevertheless, possible influences on the radiographic outcome have to be considered even with a standardized protocol. Radiographic measurements are subject to interobserver errors especially when identification of anatomic landmarks is difficult [15]. However, our measurements on full-length weight-bearing radiographs were shown to be very reliable. The interobserver reliability was “almost perfect.” The higher discrepancy between X-ray and navigation data in the preoperative setting might be due to a higher incidence of relevant deformities and flexion contractures than in the postoperative setting. As shown in Figure 4(b), the discrepancy between radiographic and navigation measurement increases with malalignment, which is consistent with previous studies that have also shown variances between radiographic and navigation measurements, especially with increased deformity [15].

Also, measurements performed by navigation system are subject to errors. Anatomic landmarks are individually selected by the surgeon so they are prone to interobserver errors [18]. Errors during the registration process have been shown to decrease with experience with the navigation system when developing a reliable registration technique [19]. Furthermore, not holding the leg in a ligament-balanced position during registration of the limb axis might also lead to incorrect measurement. Within our study, surgery was performed by a surgeon with extensive experience in CAS TKA. Another source of error is unnoticed loosening and repositioning of the reference arrays [18]. However, control of the tracker pins at the end of surgery did not reveal any loosening in our study.

Several superior aspects of the new navigation technology of the DASH system compared to existing navigation systems have been observed. Some issues associated with CAS have successfully been addressed. The additional OR time needed due to navigation is still a concern against the wider use of navigation [20] as it is associated with higher morbidity and also causes additional costs [21]. In our study, the mean duration of surgery was increased only by 5 minutes in the navigation group compared to the conventional group (78 versus 73 minutes), which is superior to established navigation systems. A meta-analysis by Bauwens et al. including 33 studies and 3423 patients showed that the use of established navigation systems extended OR time by 17 minutes (90 versus 73 minutes) [20]. Further, the concept of presenting the information in line with the working field of the surgeon leads to instant visual feedback of the surgeon's movements within the surgical field. This is facilitated by implementing an existing high-tech consumer market device (Apple iPod Touch) into the surgical procedure. The simplified user-friendly software algorithm and the intuitive handling have been shown to facilitate the computer-assisted surgical procedure even for navigation beginners [21]. This improvement in the human-machine interface has been shown to significantly reduce the additional time required during the learning curve compared to established navigation systems [21].

So far, it could not be proven that CAS results in a better clinical outcome. Some studies have shown similar functional results with CAS compared to the conventional technique [22, 23]. Lüring et al. showed no difference in WOMAC Score and KSS between the conventional and CAS group at five- to seven-year follow-up [24]. Similar KSS and range of motion after conventional and navigated TKA were reported by Hoppe et al. [25]. However, there are also studies indicating trends to better clinical outcomes with CAS. Longstaff et al. showed better clinical results and faster rehabilitation with good alignment [26], whereas CAS was shown to result in a better functional outcome in a study by Hoffart et al. [27]. Choong et al. concluded that CAS leads to an improved function and quality of life compared to the conventional technique [28]. Furthermore, a significant reduction in the rate of revision due to loosening was recently shown by de Steiger et al. [29]. Whether the use of the DASH system has an influence on the clinical outcome has not been investigated in this study.

The are some limitations of this study. One is the retrospective design, and another is the moderate number of cases. However, only consecutive cases were included. Potential measurement inaccuracies of both radiographic and navigation measurements as discussed above must be considered as limitations. As the study design was retrospective, the quality of radiographs was not particularly monitored, which resulted in a limited quality to some extent. Nonetheless, only radiographs with an acceptable quality were included. Furthermore, no clinical data have been collected, but this was not the aim of the study.

In conclusion, this study reveals very good precision for the new iPod-based navigation system DASH by Brainlab, which is similar to established navigation systems, while ruling out some of the main obstructions associated with CAS such as additional OR time.
